# The Dilemma at Gravesend

**Published:** 1920-08-07

**Authors:** C. E. Chapman

**Affiliations:** Gravesend Hospital, Kent, July 30, 1920.


*To the Editor of* The Hospital.

### The Dilemma at Gravesend.

*To the Editor of* The Hospital.

Sir,—I appreciate the direct question you ask me in this week's issue of The Hospital, and would assure you that I am fully alive to the question of an employers' subscription list; in fact, I recently commenced my campaign, and have had very good results.

Our position is, that instead of £4,000 required pre-war, we now want £10,000 per annum to carry on the work. It is difficult to forecast what our position will be at the end of the financial year, but I sincerely hope when our balance sheet is published it will prove conclusively that neither my Committee nor myself have neglected to probe every source for support.

We are in the unique position of being the first General Hospital within the entrance to the Port of London, and consequently many patients from Thames shipping are treated who would otherwise have to be taken to London hospitals. Notwithstanding this, I am often met with the response to my appeals that the shipping companies subscribe to the London hospitals and cannot assist us. As you know, we do not come within the scope of the King Edward VII. Hospital Fund.—I am, yours faithfully, C. E. Chapman.

Gravesend Hospital, Kent, July 30, 1920.

